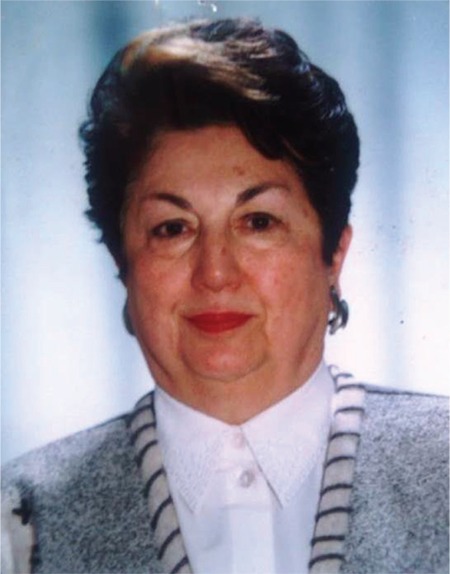# In Memory of Professor Ayhan Okçuoğlu Çavdar

**DOI:** 10.4274/balkanmedj.galenos.2019.2019.6.0001

**Published:** 2019-10-28

**Authors:** Sevgi Gözdaşoğlu

**Affiliations:** 1 Retired Professor of Pediatrics, Hematology and Oncology, Ankara University School of Medicine, Ankara, Turkey

Prof. Dr. Ayhan Okçuoğlu Çavdar passed away on June 24, 2019 at Ankara. She served as a researcher and mentor in the Department of Pediatric Hematology and Oncology at Ankara University School of Medicine for over 40 years. After her education at Erenköy Kız Lisesi, Prof. Çavdar graduated from Ankara University School of Medicine in 1953 and completed pediatric medicine residency in 1958. She received a pediatric hematology and oncology fellowship at Washington University. On returning to Turkey in 1961, she established pediatric oncology and hematology units at Ankara University for the first time in Turkey. She made several contributions to oncology in the field of leukemia, particularly orbital granulocytic sarcoma, Hodgkin’s disease, and Burkitt’s lymphoma in Turkish children. Additionally, she researched about pica syndrome in Turkey, thalassemia, hemoglobinopathies, and zinc deficiency in several conditions, especially in pregnancies and new born children with congenital abnormalities.

Dr. Çavdar wrote several research papers on these subjects that were published in international and national periodicals and books. She was elected as the first Turkish member of the Société International d’Oncologie Pediatrique - International Society of Pediatric Oncology. After receiving the Pediatric Board Certificate in 1962, Çavdar became the first Turkish member of the American Pediatric Academy. She was named as the first Turkish pediatric hematologist in the book “Hematology, the Blossoming of a Science,” written by Prof. Dr. Maxwell Myer Wintrobe in 1985. She established three research units, namely, the “Pediatric Hematology and Oncology Research Center,” “Zinc Deficiency Unit,” and “Pediatric Leukemias and Lymphomas Unit,” supported by the Scientific and Technological Research Council of Turkey (TUBITAK). She also constituted *UNESCO Satellite Trace Elements Center* in 1998. Prof. Çavdar received many awards from professional societies, including the *TUBİTAK Science Award (1976), Pediatric Oncology Service Award (1984), Prof. Dr. Nusret Fişek Public Health Service Promotion Award* (1998), and the International Network for Cancer Treatment and Research Award (2007). She published over 475 articles (approximately 197 in Turkish) and authored three books.

Dr. Çavdar defended the idea that the primary role of universities was to educate young generations and to conduct research. On attending international congresses, she shared the scientific knowledge with her colleagues. She was a prominent Figure in the department, rendering full support to us, over the years, in good and bad times. It was an honor and privilege for me to work with her.

Çavdar will always be remembered for establishing Pediatric Oncology in Turkey (1961) and as a member of The Turkish Society of Hematology (1967), Mediterranean Blood Club (1975), and The Turkish Academy of Sciences (1993). Her contributions will be greatly appreciated and will evolve where she left.

## Figures and Tables

**Figure:**